# Assessing barriers to health insurance and threats to equity in comparative perspective: The Health Insurance Access Database

**DOI:** 10.1186/1472-6963-12-107

**Published:** 2012-07-10

**Authors:** Amélie Quesnel-Vallée, Emilie Renahy, Tania Jenkins, Helen Cerigo

**Affiliations:** 1 Department of Epidemiology, Biostatistics, and Occupational Health, Purvis Hall, McGill University, 1020 Pine Avenue West, Montreal, QC H3A 1A2, Canada; 2 Department of Sociology, McGill University, Leacock Building Room 713, 855 Sherbrooke Street West, Montréal, QC H3A 2T7, Canada; 3 International Research Infrastructure on Social inequalities in health, Peterson Hall Room 328,3460 McTavish Street, Montreal, QC H3A 1X9, Canada; 4 Centre for Research on Inner City Health, St. Michael's Hospital, 30 Bond Street, Toronto, ON, M5B 1 W8, Canada; 5 Department of Sociology, Brown University, Box 1916 Maxcy Hall, 112 George Street, Providence, RI 02912, USA

## Abstract

**Background:**

Typologies traditionally used for international comparisons of health systems often conflate many system characteristics. To capture policy changes over time and by service in health systems regulation of public and private insurance, we propose a database containing explicit, standardized indicators of policy instruments.

**Methods:**

The Health Insurance Access Database (HIAD) will collect policy information for ten OECD countries, over a range of eight health services, from 1990–2010. Policy indicators were selected through a comprehensive literature review which identified policy instruments most likely to constitute barriers to health insurance, thus potentially posing a threat to equity. As data collection is still underway, we present here the theoretical bases and methodology adopted, with a focus on the rationale underpinning the study instruments.

**Results:**

These harmonized data will allow the capture of policy changes in health systems regulation of public and private insurance over time and by service. The standardization process will permit international comparisons of systems’ performance with regards to health insurance access and equity.

**Conclusion:**

This research will inform and feed the current debate on the future of health care in developed countries and on the role of the private sector in these changes.

## Introduction

In 2008, the WHO Commission on Social Determinants of Health published its landmark report stating that “inequities are killing people on a ‘grand scale’” [1]. Among the report’s recommendations for action, improving access to (public, universal) health insurance looms large, and not only in developing countries. Indeed, over the past decade, health spending in many developed countries has grown faster than gross domestic product, leading governments to search for alternative financing structures, notably through increased private expenditures [2].

However, some of the policy instruments used to reach those goals, such as restricting eligibility criteria for public insurance and increasing reliance on unregulated private health insurance (PHI) or cost sharing arrangements, may in fact have had the unexpected effect of erecting supplementary barriers to health insurance coverage. Moreover, as the impact of these policies is generally not randomly distributed in the population, these transformations have raised concerns about their effects on both population health and social inequalities in health.

**Figure 1:**
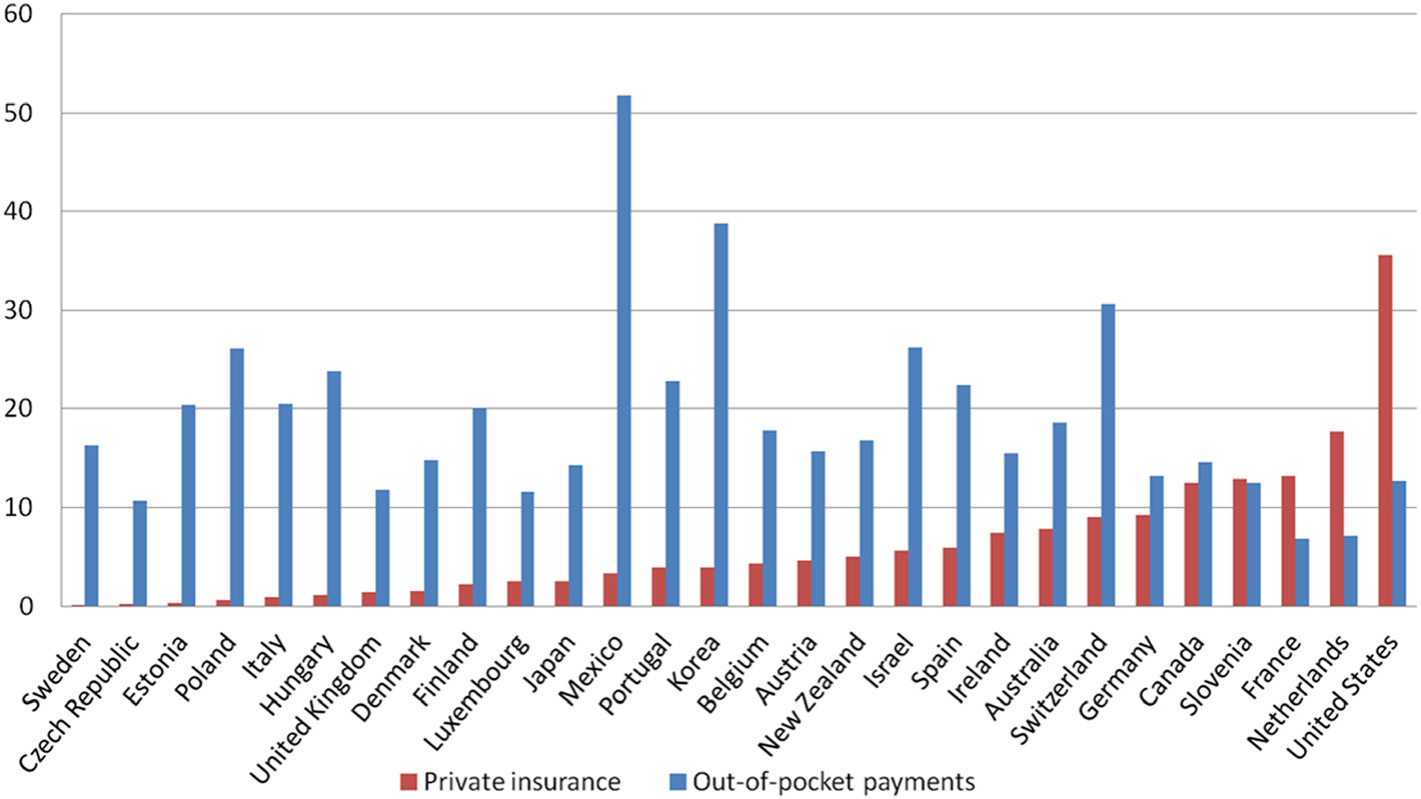
**Private health insurance spending and household Out-of-pocket payments, both as % of Total expenditures on health (TEH), OECD Countries, 2005**. *Note:* Countries are rank-ordered from left to right by private health insurance as a proportion of THE. Only countries with complete data are shown (Chile, Greece, Iceland, Norway, Slovak Republic, and Turkey omitted because of missing data on either or both variables).

For instance, in Figure 1 we present data on PHI and household out-of-pocket (OOP) spending as a proportion of total expenditures on health (TEH) among the Organisation for Economic Co-operation and Development (OECD) countries. The data are ranked by increasing proportion of PHI spending, and clearly illustrate that the proportion of OOP spending does not follow a similar upward trend. Thus, increased OOP does not necessarily follow from greater reliance on PHI. As such, we can postulate that certain countries are better able to limit financial barriers to health insurance coverage, which could occur notably because of more generous public programs, and/or through greater governmental regulation of the private insurance industry.

To examine these processes, we are developing the Health Insurance Access Database (HIAD) which assembles indicators pertaining to the interplay of public and PHI coverage and regulation. This will be achieved by extracting policy information on a set of coverage and regulation indicators in ten OECD countries, for a range of eight health services, from 1990–2010. These data will allow for the capture of policy changes over time and by service in the way health systems regulate public and private insurance. One distinguishing feature of our approach, which contrasts starkly with previous research, is a focus on the explicit and detailed measurement of policy instruments, rather than a broad typology assessment of health systems organization, as done by Roemer for instance [3,4] or the functions of PHI, as proposed by Colombo and Tapay [2]. As data collection is still underway, we present here the theoretical bases and methodology adopted, with a focus on the rationale underpinning the study instruments.

## Background

Much research has been devoted to demonstrating that health systems can improve population health. To wit, McWilliams’ [5] review of the clinical and economic literature indicates that extended health insurance coverage has indeed helped improve population health across a range of outcomes. His review, however, along with much of the research in this area, does not speak to any changes in the gap of relative inequity between the health of individuals across socioeconomic status [6-8].

In contrast, our project aims to highlight how broader, more generous and universal health insurance coverage can help narrow or widen socioeconomic gaps in health over time, often whilst improving the general health of the population [9-13]. In fact, the question of the contribution of health insurance to socioeconomic inequalities in health has received a fair amount of – mostly negative – attention since the advent of national health service systems (and particularly the British National Health Service in 1948). As it was widely assumed that social inequalities in health stemmed from unequal utilization of modern medical care, the most obvious solution to reducing these inequalities appeared to be the elimination of unequal access to medical services stemming from non-need factors – inequities, in short [14]. As such, universal health insurance and, in certain cases, universal health care systems, were the prime ways through which societies hoped to mitigate the effects of social inequalities on health [15].

Many have since argued that neither universal health coverage nor universal health care systems are sufficient for eliminating socioeconomic inequities in mortality. However, a closer examination reveals that this persistence of health inequalities is in fact often due in part to the most advantaged group taking advantage of innovations faster [16], as well as because PHI and out-of-pocket payments that are not randomly distributed in the population tend to exist alongside a public system [17-21].

### Natural experiments: Historical changes in public health insurance coverage

Most evidence comes from studies that relied upon natural experiments provided by historical changes in public health insurance coverage. One study was seminal in refining the previously popular perception that universal public health insurance in the UK had not curtailed social inequities in health. In a cross-national and longitudinal comparison of the U.K. and the Netherlands, Mackenbach, Stronks and Kunst [22] showed that the widening of socioeconomic inequities in mortality between 1930 and 1980 in the U.K. was partly due to medically amenable conditions, thus suggesting that differential access to care between socioeconomic groups may have contributed to rising socioeconomic inequities in mortality. Similar findings were reported in British Columbia, Canada [20]. More recently, a natural experiment following the introduction of universal health insurance in Taiwan found that life expectancy improved more among those in the lower classes, resulting in a narrowing of health disparities [13].

Similarly, studies that made use of the Canadian experience in developing a one-payer public system found that access to care and care of the poor both increased substantially after the introduction of national health insurance [23,24]. Moreover, it appears that this increase in access also translated in improved health outcomes: the introduction of national insurance was associated with a 4% decline in infant mortality, coupled with a decrease in the incidence of low birth weight that was particularly marked among single parents [25].

### PHI and out-of-pocket spending

An alternative perspective to these studies has been the examination of PHI and out-of-pocket spending. Longitudinal and cross-sectional studies of the effects of health insurance demonstrate a clear link between PHI coverage – especially when it is continuous – and more timely and appropriate access to care, better self-rated health and lower mortality amongst the insured [26-33]. More specifically, as they are generally unable to afford either private insurance premiums or out-of-pocket medical costs, uninsured Americans receive fewer services and have lower utilization rates than insured individuals [31,34]. Moreover, the length of time uninsured is also associated with increasing barriers to access to care [35].

### Differential access to services and social inequalities in health

Many studies have found that the likelihood of experiencing unmet health care needs is higher among people without health insurance [36-38]. Moreover, the lack of insurance also increases the probability of stating that these needs were unmet because of cost [26,36]. Both of these mechanisms could contribute to health inequalities, by increasing financial strain and decreasing access among lower SES individuals.

Recent research has also begun to focus on processes contributing to social inequalities in health by favoring the more socio-economically advantaged. This could occur for instance in mixed systems, where privately insured individuals may get access to higher quality services than their publicly or uninsured counterparts. In his overview of the literature, Bach [39] concludes that the poor are disproportionately affected by lower quality surgical care and that the gaps in quality of service experienced by different socioeconomic groups are sufficient to explain the overall gap in health between these patients. In their studies of the relationship between quality primary health care and health outcomes, Shi and colleagues [40] conclude that good primary care is not only associated with improved health, but with diminished inequities in health status between socioeconomic groups. Similarly, others found that higher quality primary health care was associated with reduced racial and ethnic disparities in both general and mental health status [41]. As such, these studies, which examine specific health services, suggest the need for a service-specific approach within a framework of social inequalities in health.

### Implications of the literature review for future research

This review indicates that, compared to public insurance, PHI and out-of-pocket spending may contribute to socioeconomic differentials in health by compounding the positive effects of income on health [26,28,42-44]. In contrast, mandatory coverage through public means should mitigate socioeconomic differentials in health as it will remove one pathway where income may impact on health [11-13,41,45]. Finally, more recent research indicates that a service-specific approach is necessary to uncover these fine-grained associations. Thus, the substantive conclusion we reach is that the mix of public coverage, private insurance, and out-of-pocket spending, especially when assessed in a service-specific perspective, can indeed potentially affect health inequalities.

Methodologically, we can also gain some directions for future research. Indeed, this review indicates that studies have rarely contrasted the experience of more than two countries, particularly in a service-specific approach. Finally, when studies contrast larger groups of countries, and/or over longer periods of time, they often have to make assumptions regarding the policy context that may have affected population health or health inequalities [46]. These considerations highlight the timeliness and highly innovative nature of the project we propose here, which will allow us to examine multiple countries and services over time using explicit, standardized measurements of policies regarding public coverage and PHI regulation.

## Development of the data collection instrument: Operational definitions

Our conceptual starting point was a taxonomy of the interplay between public and PHI proposed by the OECD [47]. Essentially, these authors argued that health insurance arrangements should be classified according to the following four criteria: Sources of financing for public or private health insurance; Level of compulsion: mandatory or voluntary health insurance; Group or individual schemes; Method of premium calculation.

Given the large number of policy instruments that fall under this classification scheme, we restricted our data collection to those most pertinent to our object, namely those that increase barriers to health insurance coverage and have the most potential for impacting health and health inequalities.

### Scoping literature review

Consequently, for each of the elements highlighted by the taxonomy, we conducted a scoping literature review to assess the published evidence regarding these insurance characteristics and their impact on both population health and health inequalities [48]. Using key electronic databases, we relied on combinations of the terms indicated in Table 1. Refined searches were performed by imposing restrictions such as geographic location (to OECD countries) and type of article (original or review). Finally, for each article that was selected, we examined its bibliography as well as later articles in which it is cited.

**Table 1:** Search terms for the scoping literature review using Web of Science and Medline

**Primary search term**	**Outcome**	**Restriction 1**	**Focus on the data collection instrument**
· Cover* or insur* or uninsured	· Health	Privat*	· Mandat*
· Insurance coverage	· inequal* OR access* OR qualit*		· Deductible
			· Criteri* AND (enrol* OR eligab*)
			· Citizen* OR residen* OR immig*
			· Income OR age-based
			· Welfare
			· Child*
			· Elderly OR seniors OR age-based
			· Disab*
			· Chronic condition/chronic disease
			· insur* OR cover* OR uninsured
			· individual health insurance OR employer health insurance OR community rated premium OR individual risk insurance coverage tax or tax break
			· Voluntary
			· Enrol*
			· Automat*
			· Application
			· Renewal
			· Premium
			· Copay*
			· Co-pay*
			· Cost-shar*
			· Duplicat*
			· Double
			· parallel
			· Complement*
			· Supplement*
			· Compuls*
			· Mandat*
			· Salutat*
			· Regulat* OR enrol* OR risk adjustment OR cream skimming
			·renew* or lifetime cover* or fee

*Notes*: These search terms were used in combinations of the four columns above, with the Boolean term AND. *General inclusion criteria*: Article type: Article and review; Language: English; Countries excluded: South Africa, Zimbabwe, Cambodia, Ghana, Kenya, Nepal, Pakistan, Tanzania, Vietnam, Slovakia, Qatar, Peru, Malaysia, Madagascar, Lebanon, Indonesia, Qatar, Saudi Arabia, Israel, Nigeria, Bahamas, Czech Republic, Ghana, India, Iran, Kenya.

**Analytic inclusion criteria:** 1) Deal with one of the topics of the question; 2) Outcome examined: inequality, access, quality, health outcomes; 3) If however, there were lack of studies on examining the outcomes, then coverage used as an outcome. **Analytic exclusion criteria:** 1) policy papers; 2) commentary; 3) proposals on how to fix the system.

In many cases, we found only indirect evidence concerning the mechanisms through which these factors could have a plausible impact on population health and/or health inequalities. This was the case for instance when studies indicated that access to health care was limited, or that certain policy configurations tended to lead to loss of coverage. We nevertheless included those indicators in our data collection instrument, as we saw this dearth of direct effects on population health or health inequalities as indicative of a knowledge gap in the literature.

### Data collection instrument

The policy instruments we focus on below were therefore those whose variation in implementation was shown or suggestive to be the most significant for population health and health inequalities. In certain cases, the broad OECD criteria had to be refined further to better reflect the range of policy instruments that we are aiming to measure here (notably to facilitate data capture by the data editors). This was the case, for instance, with the category “Sources of financing for public or private health insurance”, where we focused on private co-payments within public insurance and on sources of financing for PHI.

**Figure 2:**
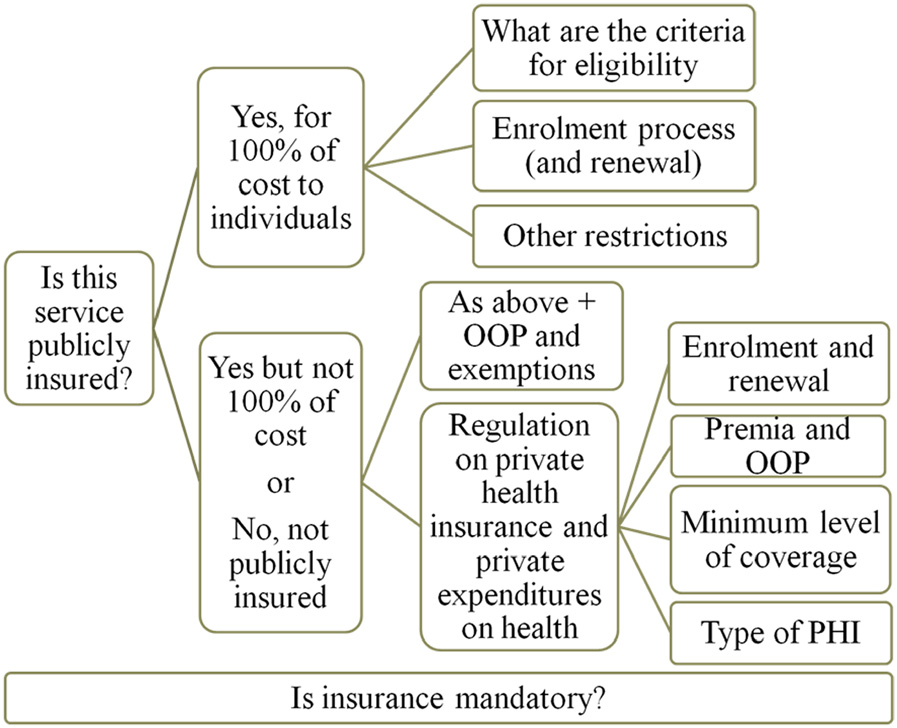
Main policy indicators collected in the HIAD.

In Table 2 we summarize the main categories of interest in the data collection instrument, provide a brief rationale for focusing on these areas of interest, and list select studies that document their potential impact on population health and health inequalities. In Figure 2, we present the policy indicators that we extract through this research process.

**Table 2:** Main HIAD indicators and their potential impact on population health and health inequalities

**Indicator or policy instrument**	**Potential impact on population health and health inequalities**	**Reference**
**Public health insurance**		
*Enrolment*	Automatic enrolment in public insurance reduces non-financial barriers to coverage (such as time-consuming, hard to understand paperwork or lack of awareness of eligibility) and increases participation rates	[49,50]
*Renewal*	The need for frequent (annually or less), active (i.e. needing action from the insuree) renewal increases the likelihood of losing coverage	[51-54]
*Cost-sharing (out-of-pocket expenses)*	Greater cost-sharing leads to decreases in service use	[55-58]
	Drug use appears particularly sensitive to this, as are economically vulnerable individuals and those with chronic diseases	[59-70].
**Private health insurance and private expenditures on health**		
*Legality of private insurance for this service*	A measure of the public prohibition of a parallel private (insurance and provision) market (see duplicative insurance below).	[2]
*Minimum level of coverage mandated by law*	The evidence suggests that a minimum coverage mandate (such as mental health parity) increases equitable access to services	[71]
*Source of financing*	Greater reliance on (unregulated) individually risk-rated insurance decreases coverage and access, but this may vary by service	[72-74]
*Tax funded subsidies*	Have a positive effect on coverage, though this may vary by service	[75,76].
*Enrolment*	Lack of regulation surrounding enrollment practices poses significant threats to coverage and access to health services	[77,78]
*Renewal*	Lifetime coverage ensures the highest levels of coverage. Low levels of public regulation increase the likelihood of lost coverage and limited access	[52,79,80]
**General mechanisms**		
*Type of coverage*	(1) Aside from strictly **public coverage**, most countries favor a mix of public and private sources for health insurance coverage.	[2]
	*PHI can be:*	
	(2) A **duplicate of public insurance**, providing a private alternative for services already covered under the public system.	
	(3) A **complement** or top-up for services already covered under the public system, as in France; (4) A supplement to public insurance for services uninsured under the public system, as in Canada;	
	(5) A **substitute to public insurance** (e.g. for those with high incomes in Germany who can opt-out);	
	(6) A **primary source of health insurance**, as in the U.S. Our preliminary results have already found evidence of other coverage types in addition to these	
*Level of compulsion for health insurance*	Mandated insurance improves access to services, but may not decrease health inequalities, unless it constitutes a mandate for public health insurance	[81,82]

As highlighted in Table 2, the first section of the data collection instrument focuses on **public health insurance.** The main questions deal with barriers to enrolment, including eligibility requirements, and with the extent of out-of-pocket payments. Our instrument explicitly attempts to document the extent to which these groups are protected from these expenses. We will also use the measures of out-of-pocket expenses provided by the OECD Health Data to supplement this indicator. The second section relates to **PHI** and private expenditures on health. A primary objective of this section is to qualify the existence of a PHI market and the extent of governmental regulation on both PHI and private expenditures on health more generally. In the last section, we seek to document the **general mechanisms** and the interplay of public and PHI in ensuring health insurance coverage of the population. The ‘type of coverage’ section was not part of the literature review because of the lack of internationally comparable studies conducted at this level – this is exactly one of the lacunae that will be addressed by our project. Our preliminary results have already found evidence of other coverage types in addition those initially mentioned by the OECD [2], such as, for instance, “complementary public health insurance”, which occurs when public health insurance covers the co-payments from PHI (an incentive that may be used by governments to increase PHI uptake).

### List of services

We drew a list of medically necessary services, using the OECD Health Data [83] as a starting point. This harmonization of the services considered in the OECD database will allow us to use these data to provide the country-level quantitative context for the qualitative policy data we are collecting. However, we also found it necessary to go beyond and encompass a broader range of medically-necessary services. Services that we added to the OECD list are indicated by an asterisk: General practitioner services; Specialist physician services (except dentists and optometry) outside hospital*; Preventive and restorative outpatient dental care services; In-patient care; Long term care; Mental health*; Prescription drugs; and Diagnostic exams and Screening.

### List of countries

This project focuses on developed, OECD countries for two reasons: 1. We rely on the OECD Health Data to quantify some of our more qualitative indicators; 2. Many analysts suggest that there is often a large discrepancy between health service legislation and its implementation in developing countries, which would bias our quantitative results towards the null [84].

**Figure 3:**
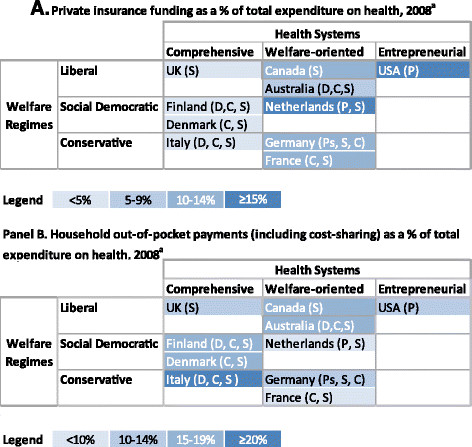
**Countries selected for analysis, by Welfare Regime and Health Systems typology by proportion of the Total expenditures on health due to private insurance funding (Panel A) and to out-of pocket payments (Panel B), and with the type of PHI in parentheses**.^a.^ Except for Denmark and the Netherlands, for which the most recent data available were in 2005. Data extracted from OECD.Stat. *Notes*: Welfare regimes are based on Esping Andersen’s typology [85]. Health systems classification is based on Roemer’s typology [3,4]. Type of PHI is based on the Colombo and Tapay OECD typology [2], and includes Primary (P), Primary substitute (Ps), Supplementary (S), Complementary (C), and Duplicative (D). Please note that some configurations of Welfare State and Health Systems typologies result in empty cells, such as for instance Social Democratic and Entrepreneurial. Finally, the Netherlands is a mixed system in many ways, and it may also be argued that it belongs in other categories, but we are relying on Esping Andersen’s and Roemer’s categorization of this country.

In a first phase, we will analyse ten countries: Australia, Canada, Denmark, Finland, France, Germany, Italy, the Netherlands, United Kingdom, and United States. These countries were selected to provide a range of variation (and thus to allow for contrasts) with regards to Esping Andersen’s Welfare Regime classification (Liberal, Social Democratic, Conservative) [85], Roemer’s type of health system (comprehensive, welfare oriented, entrepreneurial) [3,4] and the OECD’s typology of the role of PHI within that system (primary, primary substitutive, supplementary, duplicative or complementary) [2]. Figure 3 illustrates the position of countries within these different typologies, and the types of PHI that exist in these countries. A first observation is that there is some variation in the type of health system within each welfare regime (across rows), and that there also exists a variety of types of PHI within each type of health systems (across columns). Moreover, if we contrast Panel A and Panel B, we can see that the position of each country with regards to PHI (from less reliance in paler blue to greater reliance in dark blue) does not necessarily coincide with the proportion of OOP. Finally, the relative position of countries even within a given Welfare Regime/Health System cell may even be inverted with regards to the proportion of spending due to PHI or OOP: for instance of the Netherlands and Australia, where the former has a higher proportion of PHI than Australia, but a lower proportion of OOP.

### Reference period (1990–2010)

In order to track the recent evolution of health policies in many health care systems, we will be focusing on the reference years 1990, 1995, 2000, 2005, and 2010. These periods have seen frequent policy reforms with a high potential for affecting the coverage structure of health services in developed countries. On the basis of currently available evidence, we have deemed this sufficient in terms of periodicity. However, we also capture the detailed evolution over time (based on major reforms) of policy changes regarding eligibility for public coverage and exemptions from the out-of-pocket expenses associated with public coverage. Future reforms will be subsequently updated every following 5-year period for as long as possible.

### HIAD data collection strategy

**Figure 4:**
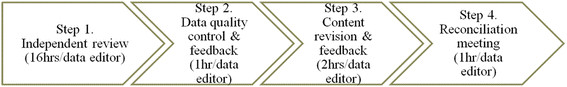
Workflow process for the individual data editors reviews.

In order to collect policy data along the dimensions we defined above, a systematic search is conducted by two data editors per case (where a case consists of a jurisdiction, a health service, and spans the entire time period of analysis) to find all relevant information using primary and secondary sources of information. We have developed a data collection instrument that standardizes the process to limit individual variations in interpretation and coding as much as possible. This standardized coding process is achieved through a thorough initial training and workflow management. This workflow is illustrated in Figure 4, which shows that each complete case takes at about 40 person-hours (this estimate does not include the time spent by the quality controller and the project manager in providing feedback respectively between steps 1 and 2 and steps 2 and 3, or the time spent by the project manager in reconciling data sheets after the reconciliation meeting).

In addition, we have trained our research team to follow the following systematic and standardized data collection process: the first citation should be a secondary source, ideally from a peer-reviewed journal, found through academic databases using a combination of MeSH terms and keywords such as those shown in Table 1. The importance of this recommendation is that this first citation should allow the data editor to narrow down the policy documents or periods that they should search for in the second citation.

The second citation should be a primary policy source, which, if recent, can often be found on websites of the countries’ Ministries of Health or health agencies. Alternatively, websites of international organizations as well as national legislative databases will be consulted, such as the WHO International Digest of Health Legislation (IDHL), The World Legal Information Institute (WorldLII), or the Foreign Law Guide (FLG).

This double citation strategy and the depth of the search for the second citation are particularly important for PHI regulation categories. Indeed, it is extremely challenging to prove the *absence* of a policy. We will therefore ensure that all necessary steps were taken to document this policy before we can affirm with any degree of certainty that there is no regulation. We describe in greater detail in the limitations section how we will code this information.

In addition to the main categorical response collected to qualify each indicator, detailed information is also recorded during the process, in a comments section or standardized research log within the data collection instrument. This “qualitative” information will be crucial to better understanding the evolution of service coverage over time and will be used for developing fine-grained categories and increasing the discriminatory power between years and/or countries.

### Data validation

The extraction, coding and standardization of policy information are delicate processes that require the implementation of careful strategies to ensure data validity and reliability [86,87]. Triangulation is used in order to verify the validity, consistency and accuracy of the results. As mentioned earlier, at least two citations are needed to justify every single item response. Moreover, in order to improve data reliability and avoid capture error or misinterpretation, we use a double rating or inter-rater reliability process: each country/service/time period is assessed by two different data editors independently. In addition, to avoid a systematic bias in data editor pairs, we regularly rotate the data editor dyad configurations.

Data editors must send in their work before a team meeting, and the project manager collates the information, highlighting any discordant information or discrepancies between raters. In the event of such problems, we discuss these issues as a team, and attempt to resolve disagreements. In the course of these discussions, priority is given to primary policy sources. In our experience so far (with 4 health services across the 10 Canadian provinces), these issues arose primarily because of the data collection instrument, and we refined it accordingly, with the consequence being that now, our raters are reaching very high levels of inter-rater reliability.

### Building and testing the HIAD

Information is currently being gathered in Microsoft Excel spreadsheets. This constitutes our raw data collection. In order to produce a database that will be functional for both qualitative and quantitative data analysis, these data are then automatically extracted from Excel into a Microsoft Access database. This database records data on all the indicators listed above, in a three-dimensional matrix: for each time period, within each jurisdiction, and for each service. Once validated, the Access database can easily be used to guide the descriptive trend analysis. It is also transferrable into a statistical software for linkage to individual-level micro-data.

### Sharing the HIAD

We have designed the HIAD to be open-access and will make the completed and validated cases available upon request. Please see our website (bit.ly/HIADaccess) for more information. In addition, we are currently examining options for online publishing that would allow for the descriptive analysis of the policy data along the dimensions of jurisdictions, time and health services.

In sum, the development of HIAD will allow for analyses along the following dimensions: within and across countries; within and across services; and within and across time periods. On that basis, there will then be countless possible combinations of these analytic dimensions, such as 1) Examining the evolution of insurance regulation of a specific service across OECD countries over several time periods; or conversely, 2) Studying how a specific country regulates the entire range of services at a specific point in time. Finally, we are building the HIAD in a way that will eventually allow for linkages with individual micro-data, in order to empirically test the effects of these different configurations of health services on inequalities of access and health.

## Limitations and sensitivity analyses

### Level of analysis

Health policy is enacted at many different levels (country; subnational; local or municipal), and the extent of this executive integration varies substantially between countries and notably as a function of the type of political system in place [88]. We therefore had to choose the most appropriate level of analysis for the database. Except for Canada (for which we are currently collecting data at the provincial level), we selected the broadest (country) level. While this broad level of analysis constitutes a potential limitation of our approach by not capturing more fine-grained local policies, we argue that it still provides a sense of the context in which these more local policies are being enacted, and plausibly sets the range, if not the minimal standard, for these policies.

### Documenting the lack of policy regulation

As we have outlined above, the careful documentation and coding of existing policies is a delicate process. This endeavour becomes even more challenging when we are trying to document the *absence* of regulation, as the burden of proof rests upon a lack of evidence. To address this issue, which is particularly pressing with PHI regulation (or lack thereof), we have developed a strict protocol that delineates the types of sources to query, in which order, for how long, and which response to record in the database at the end of the search.

Our data editors have been instructed to only select the “no regulation” category when they find two citations that explicitly state independently (i.e. without referencing each other) that there is no public regulation of PHI for this service. This is the only case where the two citations documenting our indicator can be from secondary sources (as it is unlikely that a governmental document would explicitly state that there is a *lack* of policy).

However, this stringent criterion is difficult to meet in most cases lacking regulation. Thus, barring these two citations, and when faced with a potential lack of regulation for PHI, the data editors are then instructed to search through all the sources described in the HIAD data collection strategy section above. If, after querying these sources, regulation is still lacking, this case is flagged as “no regulation found”.

### Legislation and implementation

While we could argue that it is a necessary step towards regulation, the existence of legislation does not guarantee: 1. That it will be implemented; 2. That this will be done in a timely manner; 3. That it will be respected (and enforced in cases of violation). Thus, while this project constitutes a step forward by systematically indexing the presence of policy and regulations (in contrast with a broad categorization of the system into certain health systems typologies), we still need to establish the validity of this approach with regards to the conditions above.

The periodicity of observation that we selected (every 5 years) responds in part to the second issue. Indeed, we expect that there will be a certain lag between the time legislation is passed and its implementation. In this context, we expect that five years constitutes a reasonable lag to observe the effects of a policy change.

In addition, we will perform qualitative sensitivity analyses in order to test whether the legislation we have identified meets the three conditions above. As a validation study, we will therefore perform an in-depth examination of these criteria for 3 policy changes having occurred between 1990–2005. Here, 1990 will serve as a baseline, and we will end in 2005 to ensure a sufficient lag post-legislation to observe implementation – or lack thereof.

Cases will be selected through random draw in a sequential process from the following samples:

1. All the cases of policy change in our database for the year 1995;

2. All the cases of policy change for the year 2000, excluding those coming from the same jurisdiction or for the same service as case 1;

3. All the cases of policy change for the year 2005, excluding those from jurisdictions and services from cases 1 and 2;

The stratification of samples by year is to ensure that we are observing different waves of policy change (as there tends to be diffusion of these changes from jurisdiction to jurisdiction, and “generations” of policy changes). The exclusion criteria for cases 2 and 3 similarly work to ensure that our results are not biased either by a particular political context that is more or less favourable to policy change, or by policy change for certain services where implementation may meet varying degrees of resistance, depending on the stakeholders involved (i.e. health professionals, insurance companies, etc.) and their endorsement of the policy change.

## Conclusion

This project will develop a new database standardizing the documentation of policy instruments that will provide much-needed descriptive information on the evolution and extent of public coverage and private health insurance regulation for different health services, and across many countries and time periods.

In addition to this policy analysis, the database is developed to allow for linkage with individual-level micro-data, thus setting the stage for empirical analyses of the impact of the mix of public and private health insurance on social inequalities in health and access to care. This should lead to the identification of policy-amenable contributors to health inequalities. For instance, HIAD indicators could be linked to household panel surveys (where multiple individuals from a single household are interviewed and followed over time), which would set allow for multilevel analysis at three levels of information: individual, household and country-levels. As such, contextual effect of health systems could be assessed with mixed effects models while issues of health and socioeconomic selection into health insurance coverage could be assessed with within-individual, within-family and within-country fixed effects models. Alternatively, HIAD indicators pointing to a policy change could be used with aggregate, macro data in interrupted time-series analyses to estimate the longitudinal effects of an intervention in a quasi-experimental design.

Furthermore, our open access policy with regards to sharing the HIAD data will ensure that these data are widely disseminated and used by researchers and key stakeholders. In doing so, we hope to inform and feed the current debate on the future of health care and the role of the private sector in these changes. As such, this research should lead to the development of evidence-based health care policies which are better able to protect population health and equity.

## Competing interests

The authors declare that they have no financial or non-financial competing interests.

## Authors’ contributions

AQV conceived of the study question and design, and drafted the manuscript. ER was involved in developing the research questions and the HIAD database, data collection and study coordination. TJ participated in data collection, study coordination and drafting the manuscript. HC participated in data collection, improving the HIAD database and study coordination. All authors revised the manuscript critically and approved the final manuscript.

## Pre-publication history

The pre-publication history for this paper can be accessed here:

http://www.biomedcentral.com/1472-6963/12/107/prepub
